# Association of child marriage and nutritional status of mothers and their under-five children in Bangladesh: a cross-sectional study with a nationally representative sample

**DOI:** 10.1186/s40795-024-00874-6

**Published:** 2024-05-02

**Authors:** Shanta Akter Mim, Abu Sayed Md. Al Mamun, Md. Abu Sayem, Md. Abdul Wadood, Md. Golam Hossain

**Affiliations:** Health Research Group, Department of Statistics, University of Rajshahi, Rajshahi, 6205 Bangladesh

**Keywords:** Mothers’ child marriage, Nutritional status, Under-five children, Two-level logistic regression model

## Abstract

**Background:**

Child marriage remains an important problem around the world with young mothers and their under-five children often experiencing under-nutrition. The problem is rarely studied in the Bangladeshi population. This paper was designed to identify the association between child marriage and nutritional status of mothers and their under-five children in Bangladesh.

**Methods:**

Nationally representative secondary data was used for this study, data was extracted from the Bangladesh Demographic and Health Survey (BDHS) 2017–18. The sample consisted of 7235 mothers aged 18–49 years and their under-five children. The mothers were classified into two classes according to their age at first marriage: (i) child marriage (marriage at < 18 years) and (ii) not child marriage (marriage at ≥ 18 years). The nutritional status of mothers was measured by body mass index (BMI), and under-five children’s nutritional status was measured by (i) height-for-age (z-score) (stunting), (ii) weight-for-age (z-score) (underweight), and (iii) weight-for-height (z-score) (wasting). The chi-square test and two-level logistic regression model were used for data analysis using SPSS software (IBM version 20).

**Results:**

The prevalence of child marriage among Bangladeshi women was 69.0%, with the mean and median of age at the first marriage being 16.57 ± 2.83 years and 16 years, respectively. Of the mothers, 15.2% suffered from chronic energy deficiency (underweight), and 72.8% were married at < 18 years. The prevalence of stunting, underweight, and wasting among under-five children in Bangladesh was 31.0%, 22.0%, and 8.5%, respectively. Compared to women married at the age of ≥ 18 years, there was a significantly higher likelihood of chronic energy deficiency among women who married at < 18 years [Adjusted OR = 1.27, CI: 1.05–1.82; *p* < 0.05]. Under-five children of mothers married before the age of 18 were more likely to have stunting [Adjusted OR = 1.201, CI: 1.11–1.72; *p* < 0.05], wasting [Adjusted OR = 1.519, CI: 1.15-2.00; *p* < 0.01], and underweight [Adjusted OR = 1.150, CI: 1.09–1.82; *p* < 0.05] compared to children of mothers who married at age ≥ 18.

**Conclusion:**

The rate of child marriage among Bangladeshi women is high, and it is significantly associated with malnutrition among mothers and their under-five children. The Bangladesh government can use the findings of this study to prevent and reduce child marriage and malnutrition among mothers and their under-five children to achieve sustainable development goals by 2030.

**Supplementary Information:**

The online version contains supplementary material available at 10.1186/s40795-024-00874-6.

## Introduction

A marriage before 18 years is considered a child marriage [1]. It happens in almost all nations, irrespective of ethnicity or race [2]. However, it is a social problem, particularly in low- and middle-income countries (LMICs) [1]. It threatens the life, well-being, and fundamental human rights of girls, causing a barrier to sustainable development [3]. Though both sexes face the consequences of child marriage, girls are the worst sufferers [1]. They mostly experience poor health and nutritional problems due to their specific biology and reproductive role [4]. Moreover, it has an impact on girls’ education and health, exposes them to violence, undermines prospects and potential, and traps them in poverty [1]. Global policymakers agreed to address this social problem and its consequences [5].

Child marriage is more prevalent in the South Asia region, which increases the risk of violence, violation of human rights, and deterioration of the general, sexual, and reproductive health of early married women. In some societies, girls are forced to discontinue education and marry, and once married, child girls are forced to bear and rear children [3, 6]. Some studies mentioned that child marriage was linked to poor education levels, fewer economic opportunities, and poor health among young women [7–10]. Bangladesh is one of the South Asian countries with similar social problems that have impacts on women who marry at the age of < 18 years and their offspring.

In 2013, the United Nations Children’s Fund (UNICEF) reported that 65% of Bangladeshi women married before the age of 18, and of them, 29% married before the age of 15. Though a gradual improvement was observed (around 1% per year) in 2020, 59% married before 18, and 22% married before 15 years, indicating that the social problem was not yet eliminated [11]. Bangladesh is still among the top ten countries for child marriage in the world [11]. The main causes of child marriage in the country were found to be dowry, social pressure, poverty, parents’ illiteracy [12], and some other socioeconomic and demographic factors [13–15]. Several studies in South Asia investigated the factors associated with the age at the first marriage of women and the age at the first birth and found child marriage was associated with a lower age at the first birth and higher fertility with inadequate birth spacing [16, 17]. Over time, the risks aggravate due to weak social protection mechanisms and natural factors such as floods, droughts, and COVID-19. UNICEF estimated that about 10 million girls would be at risk of becoming child brides as a result of the COVID-19 pandemic [18].

Early marriage is linked to poorer nutritional status among early pregnant women (≤ 15 years) compared to late pregnant women (≥ 19 years) [19]. In both South Asia and East Africa, child brides have been identified as a strong risk factor for stunting among under-five children [20–23]. This important issue is poorly documented in Bangladesh. However, to the best of our knowledge, four studies are available related to our present study. In the first study, the authors tried to find out the mortality trend of children and the impact of child marriage on under-5 children’s morbidity and mortality [24]. The second study investigated trends in adolescent birth and examined their associations with child under-nutrition [25]. In the third study, early childbirth and under-five malnutrition were investigated [26]. The fourth study examined child marriage and adolescent motherhood among Bangladeshi women [27]. It was clear that the study on mothers’ child marriage and the nutritional status of mothers and their under-five children was not available in the Bangladeshi population.

We therefore designed to identify the association between child marriage and malnutrition among mothers and their under-five children in Bangladesh. This study would help the government reduce the number of malnourished mothers and under-five children.

## Methods

### Study design and data

We used secondary data that was extracted from the Bangladesh Demographic and Health Survey (BDHS) 2017–2018. It was the latest cross-sectional household survey throughout the country. We used 7235 Bangladeshi adult women aged 18–49 years and their last-born under-five children as samples. The BDHS collected household, socio-demographic, lifestyle, and health-related information of mothers and their under-five children from October 2017 to March 2018. Moreover, BDHS 2017–18 measured the height and weight of the selected women and their under-five children. The study population, sample, study design, questionnaire, instruments, data collection procedure, and data reliability were described elsewhere [28].

### Inclusion criteria

Bangladeshi non-pregnant married women, living in Bangladesh, aged 18–49 years, and having at least one under-five child living with mothers (who were eligible for height and weight measurements) were considered as samples for the analysis.

### Sampling and sample selection procedure

**Fig. 1:**
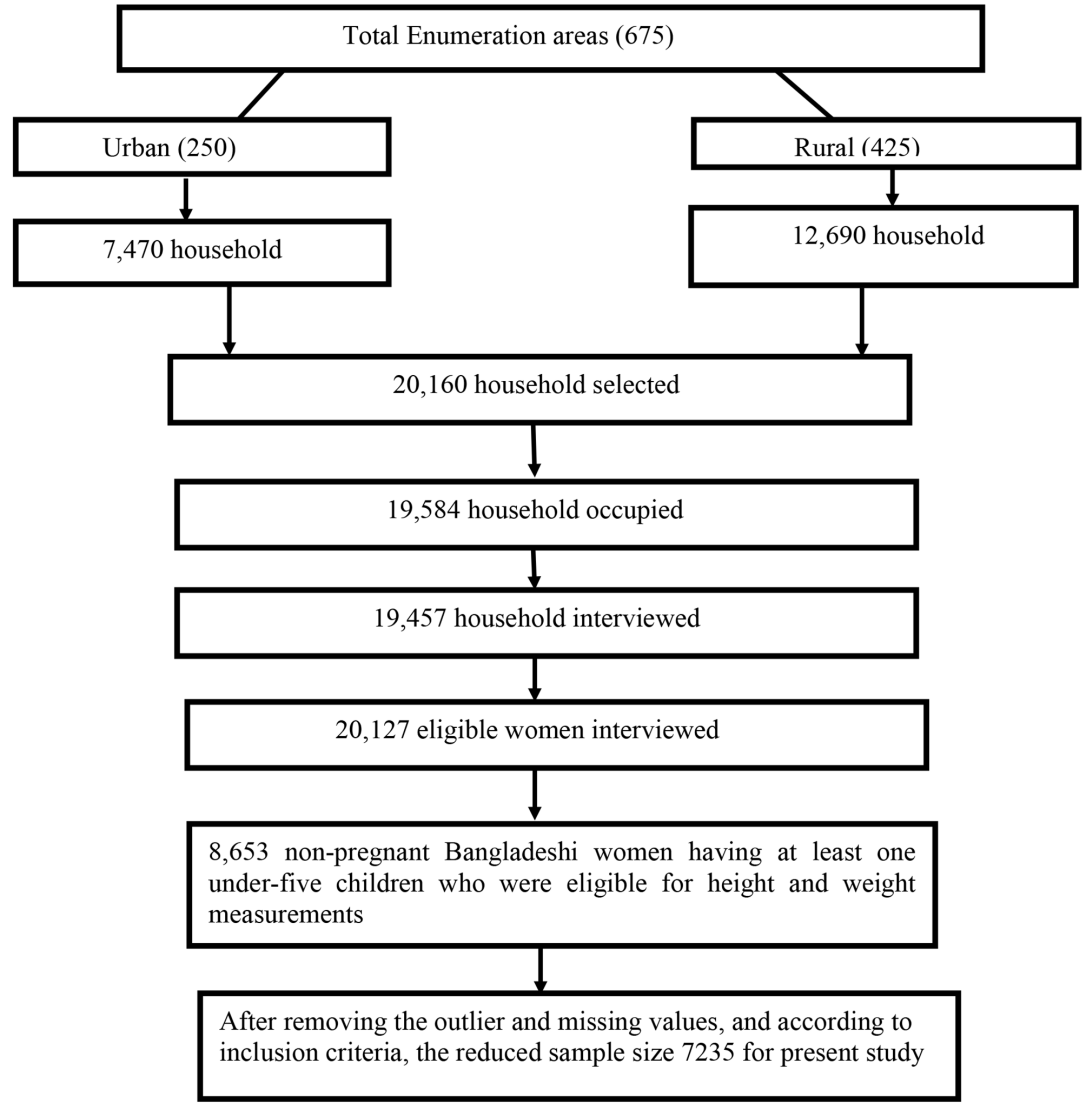
Sample selection procedure for the present study

BDHS 2017–18 used two-stage stratified cluster sampling for selecting households from Bangladesh. In the first stage, 675 enumeration areas (EAs) (250 in urban and 425 in rural areas) were selected by stratified sampling with proportional allocation. In the second stage, 30 households were selected from each selected EA using systematic sampling. BDHS 2017–18 eliminated three EAs due to communication problems and finally considered 672 EAs and 20,160 households for the survey. They mentioned that the sampling weights were not expected to lead to any significant differences in the overall survey indicators [28]. For the present study, we first considered 8,653 women with at least one under-five child who were eligible for height and weight measurements. BDHS 2017–18 considered one child if a woman had twin babies. According to our exclusion criteria, we excluded some women and their under-five children. Data were checked, and the outliers of the dataset, missing values, and incomplete data were excluded. Finally, 7235 women and their last-born under-five children were considered for the present study (Fig. 1).

### Outcome variable

There were two outcome variables for the study: (i) nutritional status of mothers, measured by their body mass index (BMI), where BMI = weight (kg) / ((height (m))^2^. Mothers were defined as having chronic energy deficiency if their BMI was < 18.5 kg/m^2^, normal weight (18.5 ≤ BMI < 25 kg/m^2^), and over-nutrition if their BMI was ≥ 25 kg/m^2^ [29]; (ii) the nutritional status of under-five children was measured by three indicators: (i) stunting (height-for-age, z-score below − 2), (ii) underweight (weight-for-age, z-score below − 2), and (iii) wasting (weight-for-height, z-score below − 2). Each indicator was classified into two classes according to the cut-off point suggested by WHO; stunting (stunted: code 1, not stunted: code 0); underweight (underweight: code 1, not underweight: code 0); and wasting (wasted: code 1, not wasted: code 0) [30].

### Independent variable

First, the main independent variable age at the first marriage (year) (AAFM) was divided into five groups; (i) AAFM ≤ 15, (ii) AAFM ≤ 16─<18, (iii) AAFM ≤ 18─≤20, (iv) AAFM ≤ 21─≤24, and (v) AAFM ≥ 25. Finally, it was divided into two groups according to the rule of the Bangladesh government: (i) child marriage (AAFM < 18 years) and (ii) not child marriage (AAFM ≥ 18 years) [28]. Child marriage was categorized as yes (code, 1), and not child marriage as no (code, 0). Some socioeconomic, demographic, and household factors were also considered independent variables in this study, as mentioned in Table 1. We followed some previous studies for selecting the variables and preparing their categories [28, 31, 32].

**Table 1:** The characteristics of selected (7235) samples (women aged 18–49 years) in Bangladesh

Variable	Group	*N* (%)	Variable	Group	*N* (%)
Division	Barisal	761(10.5)	Mothers’ education level	No education	529 (7.3)
	Chittagong	1181(16.3)		Primary	2064(28.5)
	Dhaka	1031(14.3)		Secondary	3399(47.0)
	Khulna	786(10.9)		Higher	1,243(17.2)
	Mymensingh	850(11.7)	Husbands’ education level	No education	1189 (16.4)
	Rajshahi	752(10.4)		Primary	2411(33.4)
	Rangpur	829(11.5)		Secondary	2311 (31.9)
	Sylhet	1,045(14.4)		Higher	1324 (18.3)
Type of place of residence	Urban	2,482(34.3)	Religion	Muslim	6606(91.3)
	Rural	4753(65.7)		Others	629(8.7)
Wealth Index	Poor	3022(41.8)	Total ever born children	One	2141(29.6)
	Middle	1307(18.1)		Two	2,649(36.6)
	Rich	2906(40.1)		Three or more	2,445(33.8)
Age at first birth (AAFB) (Year)	Early child bearing (AAFB < 20 years)	5005 (69.2)	Initial breastfeeding	Yes (within one hour of delivery)	3079(60.8)
	Normal (AAFB ≥ 20 years)	2230 (30.8)		No	1982(39.2)
Husbands’ occupation	Hard working	5040(69.7)	Respondent currently working	No	4262(58.9)
	Service holder	447(6.2)		Yes	2973(41.1)
	Businessman	1552(21.5)	Sex of child	Boy	3782(52.3)
	Unemployed	196(2.7)		Girl	3453(47.7)

### Statistical analysis

The background characteristics of the samples were summarized using a frequency distribution. We determined the proportion of each category of outcome variables and women’s child marriage using frequency distribution, and descriptive statistics was used to calculate the mean ± SD and median of AAFM of women and the mean ± SD of women and their under-five children age. The chi-square (χ^2^) test was utilized to examine the significance of the association between women’s child marriage and the nutritional status of women and their under-five children. The analysis of variance (ANOVA) was also used to find the variation in mean BMI among AAFM groups. As we mentioned, BDHS 2017–2018 collected data from overall Bangladesh using two-stage stratified cluster sampling; the data came from different levels of hierarchy. There was a cluster effect on the data set; a single-level statistical model would not be appropriate for analyzing this type of data set [33]. In this study, two-level logistic regression analysis was used for accounting cluster level variation and examining the association between mothers’ child marriage and the nutritional status of mothers and their under-five children. In two-level logistic regression, children/women was at the unit level and cluster at the second level. The cluster level variation was calculated using the formula mentioned in Nakagawa et al. study [34]. One of our outcome variables (women’s nutritional status) was ordinal, where ordinal logistic regression was more appropriate to analyze the data. However, the test of Parallel Lines showed that the assumption of the model was not satisfied. Alternatively, the two-level multinomial logistic regression model was used to find the association of child marriage with mothers’ nutritional status, uncontrolling/controlling the effect of selected socioeconomic, demographic, and other factors. We also used a two-level binary logistic regression model to examine the association between mothers’ child marriage and the nutritional status of their under-five children, uncontrolling/controlling the effect of the selected socioeconomic, demographic, and other factors. The variance inflation factor (VIF) was used to examine the multicollinearity problem among independent variables in logistic models; if 0 < VIF < 5, it was judged that there was no evidence of multicollinearity [35]. We did not find any multicollinearity problems among the independent variables for both multinomial and binary logistic models. The chi-square goodness-of-fit test and the Hosmer and Lemeshow test were used to examine the goodness-of-fit of multinomial and binary logistic regression models, respectively. Moreover, the accuracy of the models was checked by the receiver operating characteristic (ROC) curve. The adjusted odds ratio (AOR, OR = e^β^, where β is the regression coefficient) with a 95% confidence interval of AOR and *p*-value were utilized to interpret the results coming from logistic regression models. Statistical significance was accepted at *p* < 0.05. Statistical analyses were carried out using SPSS software (version IBM 20).

## Results

In this study, we considered 7235 non-pregnant women with at least one under-five child. The mean age of women was 26.11 ± 5.53 years, and that of under-five children was 1.94 ± 1.44 years. The highest number of women came from the Chittagong division, while the distribution of other divisions was almost the same. More than 65% of women were living in rural environments; more than 40% of women came from poor families; about 17% were higher educated and very few respondents were uneducated; and more than 16% of women’s husbands were uneducated. Nearly 70% of women delivered their first child before reaching 20 years, and more than 60% provided initial breast milk to the newborn. About 70% of women’s husbands were doing hard work, while more than 40% of women were currently working (Table 1).

The rate of child marriage among Bangladeshi mothers was about 69%, with the mean and median age at first marriage being 16.57 ± 2.83 and 16 years, respectively. We found that non-child marriage mothers had significantly (*p* < 0.01) higher BMI (23.27 ± 4.19 kg/m^2^) than child marriage mothers’ BMI (22.46 ± 3.86 kg/m^2^). It was noted that 37.6% of mothers married at ≤ 15 years, 31.4% of them married between the ages of 16 and 18, and only 2.1% married at the age of ≥ 25. The ANOVA demonstrated that the variations in mean BMI among the groups of age at first marriage were significant (*p* < 0.01), and we observed that the mean BMI increased with increasing age at first marriage (Table 2).

**Table 2:** Mean of BMI by the categories of AAFM among women

	Mean	SD	95% CI		Minimum	Maximum
			Lower bound	Upper bound		
Age at first marriage (years)	16.57	2.83	16.50	16.63	10	36
Mean of BMI for each category of AAFM						
Child marriage (AAFM < 18 year) (4991(69.0%)	22.46	3.86	22.35	22.57	15.01	39.45
Not-child marriage (AAFM ≥ 18 year) (2244, 31.0%)	23.27	4.19	23.10	23.44	15.12	38.78
T-test value = 6.950					*p*-value = 0.001	
AAFM < 16 (2723, 37.6%)	22.45	3.85	22.35	22.63	15.01	38.14
16 ≤ AAFM ≤ 18 (2268, 31.4%)	22.47	3.88	22.27	22.58	15.01	39.45
AAFM > 18(2244, 30.9%	23.28	4.09	22.68	23.90	15.12	38.78
F-test value = 82.457					*p*-value = 0.001	

N.B: AAFM: Age at first marriage

It was noted that 15.2% of mothers were suffering from chronic energy deficiency (BMI < 18 kg/m^2^); of them, 72.8% got married before the age of 18, while the prevalence of over-nourished (BMI ≥ 25 kg/m^2^) mothers was 26.1%. A decreasing tendency in the rate of child marriage was observed with the increase in the nutritional status of mothers, and the Chi-square test showed that the association between these two factors was highly significant (*p* < 0.001). We found that the current prevalence of stunting, underweight, and wasting of under-five children was 31%, 22%, and 8.5%, respectively, and of them, 73.2%, 71.8%, and 69.8% of children’s mothers got married before the age of < 18. The association between mothers’ child marriage and under-five children’s stunting and underweight was highly significant (*p* < 0.001), while wasting was not significant (*p* > 0.05) (Table 3).

**Table 3:** Association between women’s child marriage and nutritional status of mothers and their under-five children

Indicator		*N* (%)	Child marriage		Chi-square value	*p*-value
			No	Yes		
Mothers’ nutritional status	Chronic Energy Deficiency (BMI < 18.5	1097 (15.16)	298(27.2%)	799(72.8%)		
	Normal (18.5 ≤ BMI < 25 kg/m^2^)	4244 (58.66)	1228(28.9%)	3016(71.1%)	58.255	0.001
	Over nutrition (BMI ≥ 25 kg/m^2^)	1894 (26.18)	718(37.9%)	1176(62.1%)		
**Child’s nutritional status**						
Stunting	Yes (Z-score<-2), 2245(31.0%)	2245 (31.03)	602(26.8%)	1643(73.2%)	26.846	0.003
	No (Z-score≥-2), 4990(69.0%)	4990 (68.97)	1642(32.9%)	3348(67.1%)		
Underweight	Yes (Z-score<-2), 1590(22.0%)	1590 (21.98)	449(28.2%)	1141(71.8%)	7.344	0.008
	No (Z-score≥-2), 5645(78.0%)	5645 (78.02)	1795(31.8%)	3850(68.2%)		
Wasting	Yes (Z-score<-2), 612(8.5%)	612 (8.46)	185(30.2%)	427(69.8%)	0.194	0.779
	No (Z-score≥-2), 6623(91.5%)	6623 (91.54)	2059(31.1%)	4564(68.9%)		

**Fig. 2:**
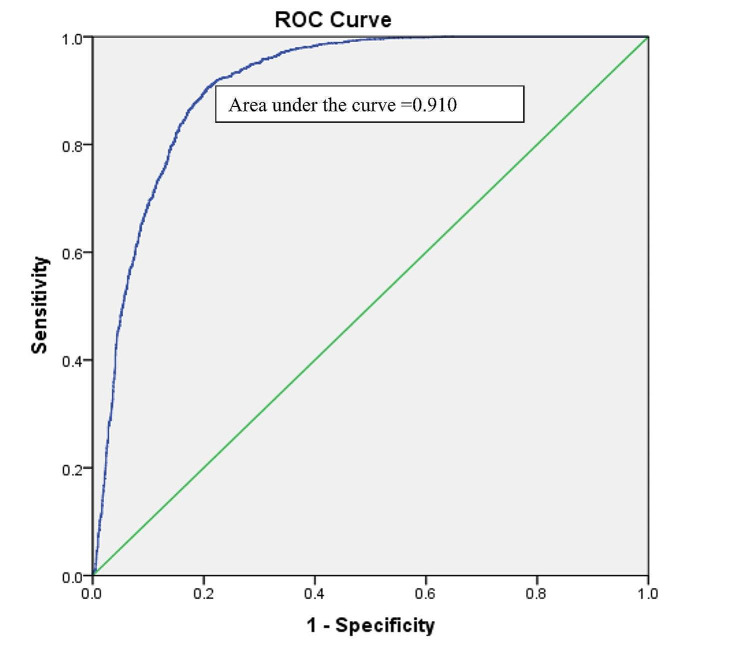
ROC curve for accuracy of multinomial logistic model

The VIF values lay between 0 and 2 indicating that there was no evidence of a multicollinearity problem among the predictor variables of the two-level multinomial model. The model demonstrated that after controlling the effect of other selected variables, a significantly higher likelihood of chronic energy deficiency was found among mothers who married at < 18 compared to mothers who married at ≥ 18. Division (living location), type of place of residence, wealth index, total ever-born children, and husbands’ occupation were the other predictors of chronic energy deficiency among mothers compared to normal weight. On the other hand, division (living location), mothers’ education level, husbands’ education level, religion, wealth index, total ever-born children, age at first birth, initial breastfeeding, and respondents’ currently working status were predictors of chronic energy deficiency compared to over-nutrition. The Pearson chi-square test showed that the two-level multinomial logistic model was good fitted to the data, and the Nagelkerke R^2^ value demonstrated that the model can explain the variation of the outcome variable by 40%. Moreover, the area under the ROC curve demonstrated that the accuracy of the model was very high (91.0%) (Table 4; Fig. 2).

**Table 4:** Two-level multinomial logistic regression analysis of child marriage influencing women’s nutritional status (chronic energy deficiency as reference case)

	Variable	AOR (95% CI: lower-upper)	*p*-value		AOR (95% CI: lower-upper)	*p*-value
Normal weight	**Child marriage**			Overnutrition		
	No	1.14 (0.91–1.42)	0.056		1.27 (1.05–1.82)	0.023
	Yes^R^					
	**Division**					
	Barisal	1.46 (1.08–1.97)	0.014		2.38 (1.60–3.55)	0.001
	Chittagong	2.31(1.71–2.07)	0		4.12 (2.85–5.95)	0.001
	Dhaka	1.54(1.15–2.07)	0.004		2.68 (1.15–3.87)	0.001
	Khulna	1.63 (1.18–2.26)	0.003		2.79 (1.85–4.22)	0.001
	Mymensingh	1.20 (0.92–1.59)	0.179		1.68 (1.15–2.47)	0.007
	Rajshahi	1.75 (1.27–2.42)	0.001		2.66 (1.75–4.06)	0.001
	Rangpur	1.55 (1.14–2.09)	0.005		2.54 (1.69–3.80)	0.001
	Sylhet^R^					
	**Type of place of residence**					
	Urban	0.80 (0.67–0.97)	0.024		1.06 (0.85–1.33)	0.59
	Rural^R^					
	**Mothers’ education level**					
	No education	0.71 (0.47–1.09)	0.118		0.49 (0.29–0.87)	0.014
	Primary	0.72 (0.52–1.01)	0.054		0.65 (0.44–0.97)	0.035
	Secondary	0.96 (0.71–1.29)	0.777		0.99 (0.71–1.39)	0.955
	Higher^R^					
	**Husbands’ education level**					
	No education	0.88 (0.60–1.27)	0.497		0.50 (0.32–0.79)	0.003
	Primary	0.87 (0.62–1.22)	0.421		0.51 (0.35–0.76)	0.001
	Secondary	0.95 (0.69–1.32)	0.799		0.70 (0.48–1.01)	0.053
	Higher^R^					
	**Religion**					
	Muslim	1.20 (0.91–1.58)	0.194		1.48 (1.13–2.12)	0.033
	Others^R^					
	**Wealth Index**					
	Poor	0.57 (0.46–0.73)	0.001		0.18 (0.14–0.25)	0.001
	Middle	0.67 (0.52–0.86)	0.002		0.41 (0.30–0.55)	0.001
	Rich^R^					
	**Total ever born children**					
	One	0.53 (0.43–0.66)	0.001		0.23 (0.18–0.30)	0.001
	Two	0.93 (0.77–1.15)	0.541		0.65 (0.52–0.84)	0.001
	Three or more^R^					
	**Age at first birth (Year)**					
	Early child bearing (age < 20)	0.89(0.72–1.12)	0.324		0.52 (0.39–0.68)	0.001
	Normal (age ≥ 20)^R^					
	**Initial breastfeeding**					
	Yes	0.89 (0.75–1.05)	0.161		0.81 (0.66–0.99)	0.035
	No^R^					
	**Husbands’ occupation**					
	Hard working	1.34 (0.82–2.19)	0.24		1.12 (0.59–2.13)	0.711
	Service holder	1.85 (0.93–3.69)	0.08		1.51 (0.67–3.40)	0.315
	Businessman	1.84 (1.09–3.12)	0.021		1.73 (0.89–3.37)	0.106
	Unemployed^R^					
	**Respondent currently working**					
	No	1.15 (0.97–1.293)	0.136		1.32(1.07–1.63)	0.01
	Yes^R^					
	**Sex of child**				.	
	Boy	1.07(0.92–1.23)	0.344		1.10 (0.91–1.33)	0.327
	Girl^R^					
	**Goodness-of-Fit**, Pearson Chi-Square Value = 3575.582				**p-value =** 0.555	
	**Nagelkerke R**^**2**^**–Value** = 0.399					

N.B: R: Reference category; AOR: Adjusted odds ratio; CI: Confidence interval

**Fig. 3:**
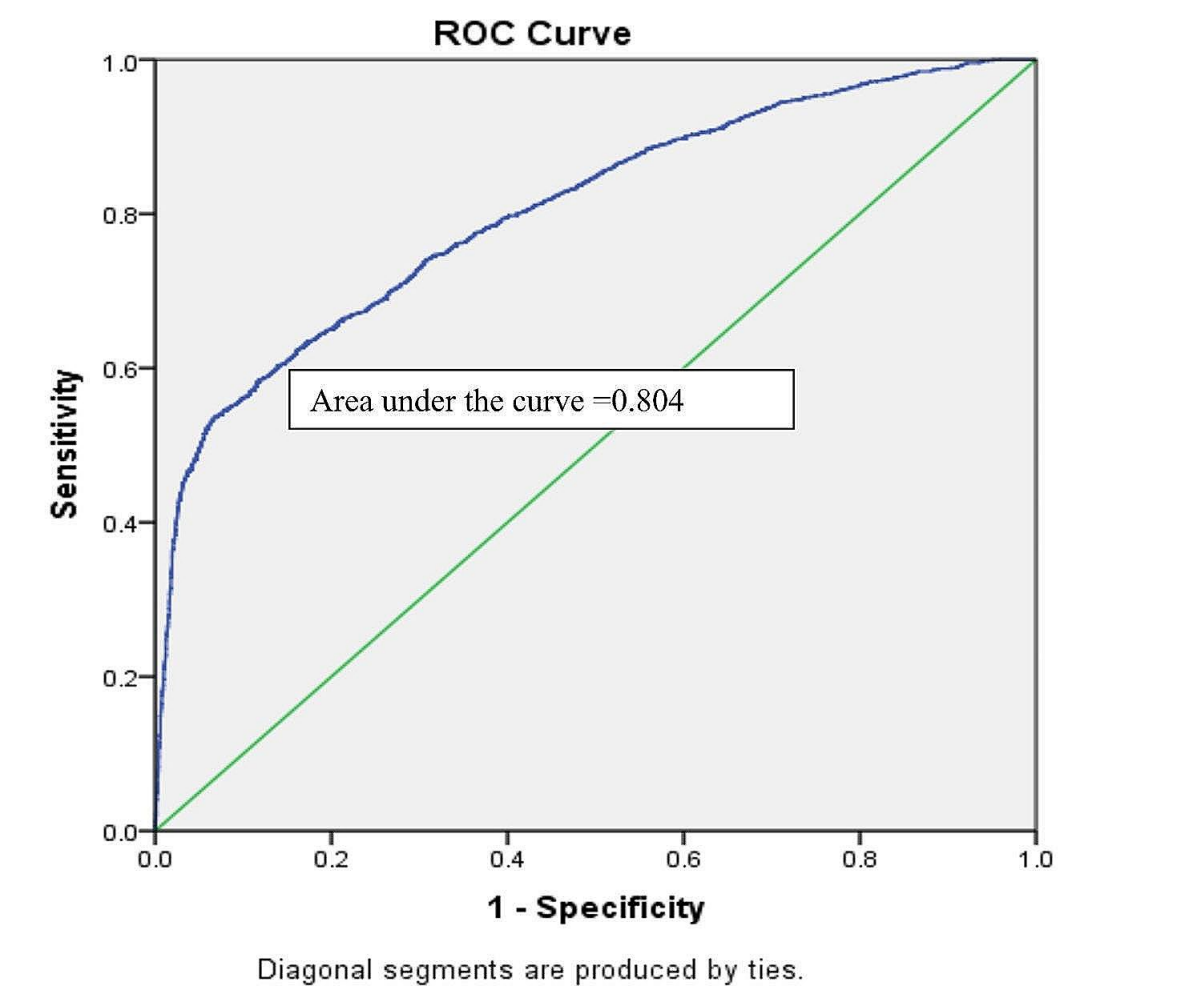
ROC curve for accuracy of binary logistic model for stunting

**Fig. 4:**
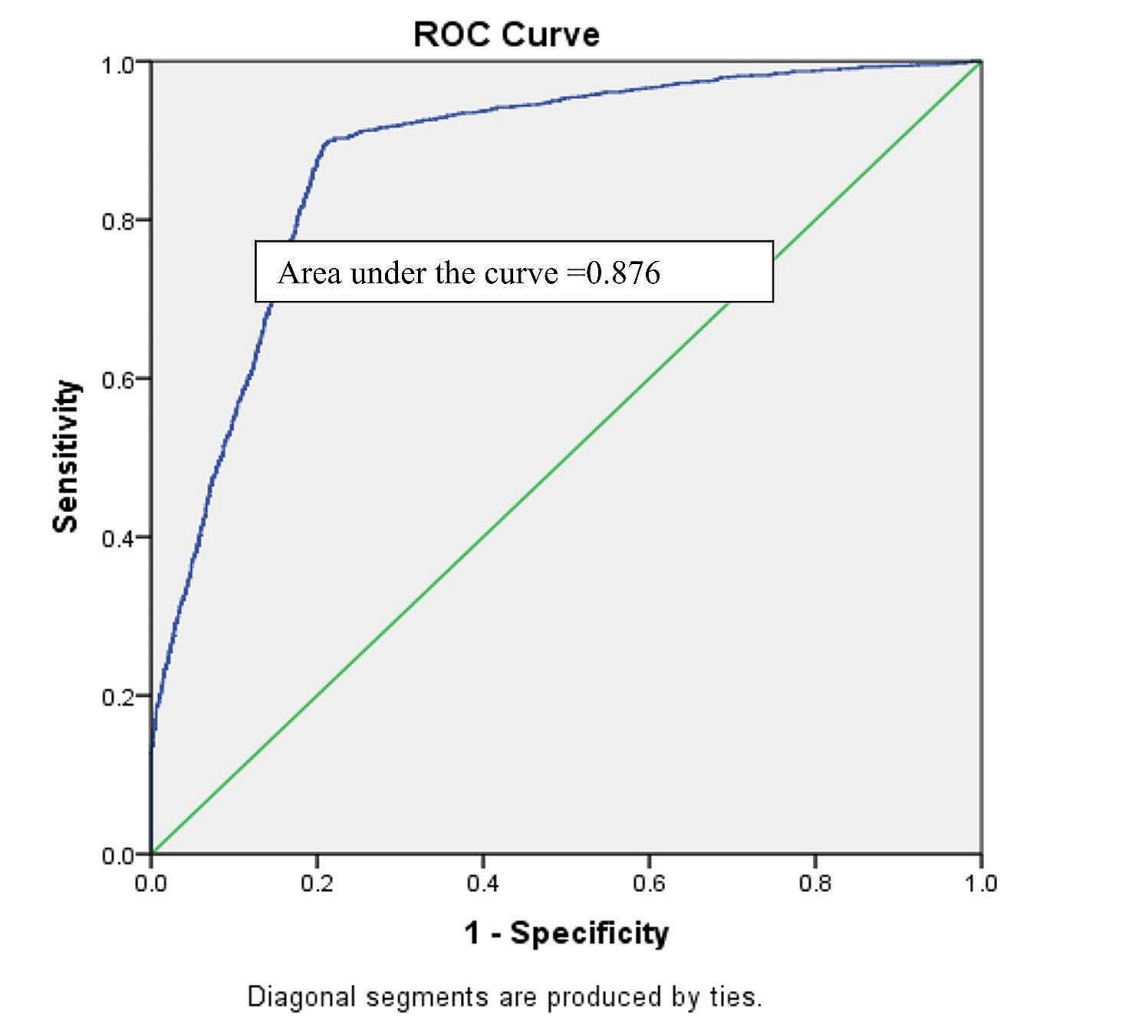
ROC curve for accuracy of binary logistic model for underweight

**Fig. 5:**
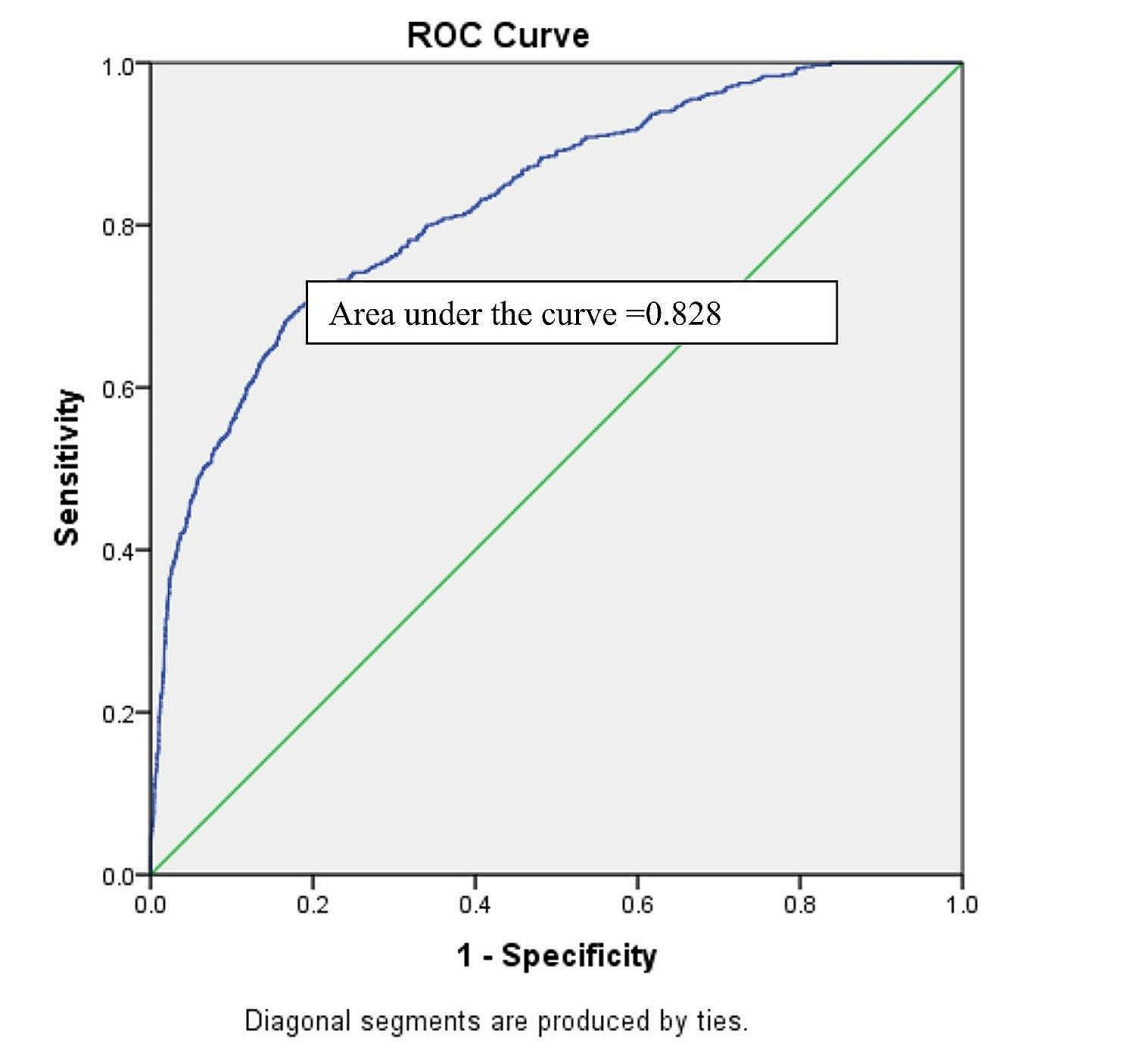
ROC curve for accuracy of binary logistic model for wasting

In case of the two-level binary model, the VIF values lay between 0 and 3 indicating that there was no evidence of a multicollinearity problem among the independent variables in the model. The model showed that after controlling for the effect of other selected variables, mothers who married before 18 were more likely to have stunted, wasted, and underweight children compared to mothers who married at the age of ≥ 18. Other important predictors of stunting and underweight among under-five children were division (living location), mothers’ education level, fathers’ education level, and wealth index. On the other hand, the mother’s education, total ever born children, and age at first birth of the mother were the significant associated factors of under-five children wasting. Hosmer and Lemeshow test showed that our selected binary logistic model was good fitted to stunting, underweight, and wasting. Nagelkerke R^2^ value showed the model can explain the variation of the outcome variables stunting, underweight, and wasting by 56%, 60%, and 49%, respectively (Table 5). Moreover, the area under the ROC curve demonstrated that the accuracy of the model was 80.4% (Fig. 3), 87.6% (Fig. 4), and 82.8% (Fig. 5) for stunting, underweight, and wasting, respectively. Detailed results are shown in supplementary Tables 1, 2, and 3.

**Table 5:** Two-level binary logistic regression analysis of maternal child marriage influencing nutritional status of under-five children

Variable	Stunting, AOR (95% CI)	Wasting, AOR (95% CI)	Underweight, AOR (95% CI)
**Child marriage**			
No	1.201 (1.11–1.72)*	1.519 (1.15-2.00)**	1.150 (1.09–1.82)*
Yes^R^			
**Division**			
Barisal	0.706 (0.54–0.92)**	0.980 (0.64–1.48)	0.636 (0.46–0.86)**
Chittagong	0.777 (0.61–0.97)*	0.888 (0.61–1.27)	0.668 (0.51–0.87)**
Dhaka	0.576 (0.44–0.74)**	0.922 (0.62–1.35)	0.512 (0.38–0.68)**
Khulna	0.574 (0.43–0.75)**	0.742 (0.47–1.17)	0.595 (0.43–0.82)**
Mymensingh	0.829 (0.64 − 0.106)	0.949 (0.64–1.40)	0.821 (0.62–1.08)
Rajshahi	0.667 (0.50–0.87)**	0.675 (0.42–1.07)	0.593 (0.43–0.81)**
Rangpur	0.599 (0.45–0.78)**	0.819 (0.53–1.26)	0.649 (0.47–0.88)**
Sylet^R^			
**Type of place of residence**			
Urban	1.031 (0.88–1.20)	1.182 (0.92–1.50)	1.131 (0.94–1.35)
Rural^R^			
**Mothers’ education level**			
No education	1.557 (1.10–2.19)**	2.071 (1.20–3.55)**	2.384 (1.60–3.53)**
Primary	1.564 (1.19–2.04)**	1.333 (0.85–2.07)	1.806 (1.31–2.48)**
Secondary	1.422 (1.12–1.80)**	1.415 (0.96–2.07)	1.592 (1.19–2.11)**
Higher^R^			
**Husbands’ education level**			
No education	1.814 (1.34–2.44)**	1.260 (0.78–2.01)	1.550 (1.09–2.19)*
Primary	1.817 (1.39–2.36)**	0.897 (0.58–1.36)	1.506 (1.10–2.05)**
Secondary	1.33 (1.03–1.71)**	1.09 (0.74–1.62)	1.266 (0.93–1.70)
Higher^R^			
**Religion**			
Muslim	0.960 (0.75–1.121)	1.236 (0.81–1.187)	0.982 (0.74–1.29)
Others^R^			
**Wealth Index**			
Poor	1.494 (1.25–1.78)**	1.115 (0.83–1.49)	1.360 (1.10–1.67)**
Middle	1.481 (1.22–1.79)**	0.963 (0.69–1.33)	1.387 (1.11–1.73)**
Rich^R^			
**Total ever born children**			
One	1.066 (0.89–1.26)	1.44 (1.09–1.92)**	0.967 (0.79–1.17)
Two	0.909 (0.78–1.06)	1.22 (0.94–1.58)	0.890 (0.74–1.06)
Three or more^R^			
**Age at first birth (Year)**			
Early child bearing (age < 20)	1.113 (0.93 − 0.88)	1.625 (1.21–2.18)**	1.196 (0.97–1.46)
Normal (age ≥ 20)^R^			
**Husbands’ occupation**			
Hard working	0.918 (0.59–1.40)	1.270 (0.62–2.60)	0.764 (0.48–1.21)
Service holder	0.815 (0.46–1.42)	0.748 (0.29–1.90)	0.689 (0.36–1.28)
Businessman	0.813 (0.52–1.27)	0.856 (0.40–1.81)	0.597 (0.36–0.97)*
Unemployed^R^			
**Respondent currently working**			
No	0.902 (0.78–1.03)	0.993 (0.79–1.24)	0.968 (0.82–1.13)
Yes^R^			
**Sex of child**			
Boy	1.08 (0.95–1.122)	1.199 (0.97–1.47)	1.139 (0.98–1.31)
Girl^R^			
**Initial breastfeeding**			
Yes	1.00 (0.88–1.14)	0.982 (0.79–1.21)	0.983 (0.84–1.14)
No^R^			
**Hosmer and Lemeshow Test**	Chi-square value = 5.843; *p*-value = 0.665	Chi-square value = 3.999; *p*-value = 0.857	Chi-square value = 6.793; *p*-value = 0.559
**Nagelkerke R** ^**2**^ **–value**	0.56	0.49	0.60
**Cluster level variation**	0.0280	0.025404	0.0752

N.B: R: Reference category, AOR: Adjusted odds ratio; CI: Confidence interval; **: 1% (*p* < 0.01) and *: 5% (*p* < 0.05) level of significance

## Discussion

We investigated the association between child marriage and the nutritional status of mothers and their under-five children, controlling for other factors. We found 69% of women aged 18–49 married before the age of 18. Child marriage was more than twice as high in rural areas compared to urban areas. However, BDHS 2017–18 reported that the percentage was 59% among women aged 20–24 in the country [28]. The proportion of child marriage has been decreasing during the last two decades in Bangladesh due to increasing women’s education [28]. BDHS 2017-18 reported that the median age of first marriage among women aged 20–49 in Bangladesh has been a slow but steady increase over time, from 14.4 years in 1993–94 to 15.3 years in 2007 and 16.3 years in 2017–18, and married by age 18 (the legal age of marriage for women in Bangladesh) has declined from 73% in 1993–94 to 65% in 2011 and 59% in 2014 and 2017–18 [28]. Still, the rate of child marriage in Bangladesh is the third highest in the world [36]. In Bangladeshi culture, marriage of aged girls is comparatively difficult, and parents here usually favor child marriage due to poverty and a lack of security. The crisis of employment for educated women is also an important reason behind it. All these factors contribute to the significantly higher rate of child marriage in the country [37]. On the other hand, our study showed that the prevalence of chronic energy deficiency among married women aged 18–49 with at least one under-five child was 15.2%, while 26.1% were over-nourished. However, the overall prevalence of under-nutrition and over-nutrition was 12% and 32%, respectively, among the same age group, irrespective of having under-five children [28]. The child marriage rate of Bangladeshi women has steadily decreased during the last two decades. At the same time, the prevalence of under-nutrition among women decreased from 52% in 1996–97 to 30% in 2007 and 12% in 2017–18, while over-nutrition sharply increased from 3% in 1996–97 and 12% in 2007 to 32% in 2007–18 [28]. There is evidence of the dual burden of malnutrition among married women aged 15–49 years in Bangladesh. The prevalence of stunting, underweight, and wasting among under-five children in Bangladesh has also been improving during the last two decades [28]. The percentage of under-five stunted children declined from 43% in 2007 to 31% in 2017-18, while the percentage of underweight fell from 41% in 2007 to 22% in 2017-18, and wasting declined from 14% in 2007 to 8% in 2017-18 [28]. This may be due to an increase in the women’s education and household wealth index in the country during the period [28]. Timing and circumstances of age at first marriage have profound consequences for women’s and men’s lives, and their effects on their health outcomes are significant. In the present study, we found a great number of women married at < 18 were suffering from chronic energy deficiency than their counterparts [38]. Child marriage was one of the most important indicator of chronic energy deficiency among married women in Bangladesh. The same type of finding was revealed in different countries around the world [38]. The variation in malnutrition of mothers and their under-five children were found among the divisions. The prevalence of chronic energy deficiency was found to be highest among mothers and children living in Sylhet division, same result had been found in a previous Bangladeshi study [39]. Some possible reasons were: the overall literacy rate in Sylhet division was lowest in the country [40], and neonatal mortality, postneonatal mortality and infant mortality rate were highest in the division [41]. Also, it was reported that lower contraceptive prevalence and higher total fertility rate in Sylhet division compared to other divisions in Bangladesh [28].

In the present study, we found that mothers’ child marriage was an important predictor of stunting and underweight in under-five children. Similar results were found in sub-Saharan African [21] and Indian studies [23, 24]. Stunting is the chronic consequence of energy deficiency from the period of pregnancy to the age of under-five with inappropriate feeding practices. This chronic deficiency can even cause some non-communicable diseases, such as heart disease, in the late ages of today’s children [42]. Child marriage mostly happens in families of low socio-economic status where health awareness, adequate nutrition, and medical facilities are scarce. This contributes to malnutrition in both child-married mothers and their under-five children [38].

In Bangladesh, only 18.2% of women are highly educated, and education is an important factor in women’s age at the first marriage. Educated mothers are usually more conscious of their and their child’s health and well-being and understand well the importance of antenatal (ANC) and postnatal care (PNC), which can prevent chronic energy deficiency among under-five children. Subsequently, more than 67% of women are from poor and middle-income groups and are at risk of getting inadequate nutritious food and fruits, thus augmenting the vicious cycle of malnutrition among mothers and under-five children [28]. Though Bangladesh has a law against child marriage, it is still an important social problem all over the country due to social culture and poor implementation of the law.

### Strength and limitations of the study

This is the first time we attempted to investigate the association between mothers’ child marriage and the nutritional status of mothers and their under-five children in Bangladesh. As this study covers all divisions of Bangladesh with a sufficient sample size, it claims strong scientific strength. In addition, appropriate steps, processes, and measurements were taken by a group of skilled data collectors who were properly trained in data ethics to avoid bias and ensure a transparent survey through the sharing of survey objectives. However, there were some limitations to our study. Firstly, due to the cross-sectional study, we could not determine the causal relationship between child marriage and the nutritional status of mothers and their under-five children. Secondly, we could not include all the factors for the multivariable model known to be related to child marriage and the nutritional status of mothers and their under-five children due to the secondary nature of the data. More research is required regarding child marriage and the health and nutritional status of mothers and their under-five children in Bangladesh.

## Conclusions and recommendations/policy implication

In this study, we found that the rate of child marriage among Bangladeshi women was high, and it had a significant association with the nutritional status of mothers and their under-five children. This study used a nationally representative sample, and the findings can be considered to revise the health policy to reduce the rate of child marriage as well as to improve the nutritional status of women and their under-five children in Bangladesh. The government health authorities, as well as non-government social and cultural organizations, should use the findings of this study and play an important role in undertaking interventions to reduce the adverse consequences of child marriage. For reducing/preventing child marriage, this study recommends (i) raising awareness among people about the disadvantages of child marriage and (ii) increasing women’s education level, women’s empowerment, and their well-being.

### Electronic supplementary material

Below is the link to the electronic supplementary material.

Supplementary Material 1

## Data Availability

Data are freely available at https://dhsprogram.com/data/dataset/Bangladesh_Standard-DHS_2017.cfm?flag=0.

## Abbreviations

AAFM: Age at first marriage
AOR: Adjusted Odds Ratio
ANC: Antenatal care
BDHS: Bangladesh Demographic and Health Survey
BMI: Body mass index
COVID-19: Coronavirus disease-19
IBM: International Business Machines
LMICs: Low- and middle-income countries
MOR: Median odds ratio
OR: Odds ratio
PNC: Postnatal care
SE: Standard error
SPSS: Statistical Package for the Social Sciences
UNICEF: United Nations Children’s Fund
