# Supplementary material for: Association of child marriage and nutritional status of mothers and their under-five children in Bangladesh: a cross-sectional study with a nationally representative sample
Source: BMC Nutr. 2024 May 2;10:67. doi: 10.1186/s40795-024-00874-6 (PMC11067235; doi:10.1186/s40795-024-00874-6)
Supplement: Supplementary file 1 — Supplementary Material 1 [file 40795_2024_874_MOESM1_ESM.docx]

**Supplementary Table 1:** Two-level binary logistic regression analysis of maternal child marriage influencing stunting of under-five children

| **Variable** | **OR** | **SE** | **P-value** | **95% CI for OR** | |
| --- | --- | --- | --- | --- | --- |
|  |  |  |  | **Lower** | **Upper** |
| **Age at first marriage (year)** | | | |  |  |
| Non-child marriage Vs Child marriage^R^ | 1.201 | 0.099 | 0.045 | 1.11 | 1.72 |
| **Division** | | | |  |  |
| Barisal Vs Sylhet^R^ | 0.706 | 0.094 | 0.009 | 0.54 | 0.91 |
| Chittagong Vs Sylhet^R^ | 0.777 | 0.090 | 0.031 | 0.61 | 0.97 |
| Dhaka Vs Sylhet^R^ | 0.576 | 0.074 | 0.000 | 0.44 | 0.74 |
| Khulna Vs Sylhet^R^ | 0.574 | 0.081 | 0.000 | 0.43 | 0.75 |
| Mymensingh Vs Sylhet^R^ | 0.829 | 0.104 | 0.136 | 0.64 | 1.06 |
| Rajshahi Vs Sylhet^R^ | 0.667 | 0.092 | 0.004 | 0.50 | 0.87 |
| Rangpur Vs Sylhet^R^ | 0.599 | 0.081 | 0.000 | 0.45 | 0.78 |
| **Type of place of residence** | | | |  |  |
| Urban Vs Rural^R^ | 1.031 | 0.0811 | 0.691 | 0.88 | 1.20 |
| **Mothers’ education level** | | | |  |  |
| No education Vs Higher^R^ | 1.557 | 0.274 | 0.012 | 1.10 | 2.19 |
| Primary Vs Higher^R^ | 1.564 | 0.213 | 0.001 | 1.19 | 2.04 |
| Secondary Vs Higher^R^ | 1.422 | 0.171 | 0.003 | 1.12 | 1.80 |
| **Husbands’ education level** | | | |  |  |
| No education Vs Higher^R^ | 1.814 | 0.274 | 0.000 | 1.34 | 2.44 |
| Primary Vs Higher^R^ | 1.817 | 0.243 | 0.000 | 1.39 | 2.36 |
| Secondary Vs Higher^R^ | 1.333 | 0.170 | 0.024 | 1.03 | 1.71 |
| **Religion** | | | |  |  |
| Muslim Vs Others^R^ | 0.960 | 0.116 | 0.739 | 0.75 | 1.21 |
| **Wealth Index** | | | |  |  |
| Poor Vs Rich^R^ | 1.494 | 0.136 | 0.000 | 1.25 | 1.78 |
| Middle Vs Rich^R^ | 1.481 | 0.145 | 0.000 | 1.22 | 1.79 |
| **Total ever born children** | | | |  |  |
| One Vs Three or more^R^ | 1.066 | 0.093 | 0.463 | 0.89 | 1.26 |
| Two Vs Three or more^R^ | 0.909 | 0.071 | 0.229 | 0.78 | 1.06 |
| **Age at first birth (Year)** | | | |  |  |
| Early child bearing (age<20) Vs Normal (age≥20)^R^ | 1.113 | 0.099 | 0.229 | 0.93 | 0.88 |
| **Husbands’ occupation** | | | |  |  |
| Hard working Vs Unemployed^R^ | 0.918 | 0.200 | 0.697 | 0.59 | 1.40 |
| Service holder Vs Unemployed^R^ | 0.815 | 0.231 | 0.473 | 0.46 | 1.42 |
| Businessman Vs Unemployed^R^ | 0.813 | 0.184 | 0.364 | 0.52 | 1.27 |
| **Respondent currently working** | | | |  |  |
| No Vs Yes^R^ | 0.902 | 0.062 | 0.140 | 0.78 | 1.03 |
| **Sex of child** |  |  |  |  |  |
| Boy Vs Girl^R^ | 1.08 | 0.068 | 0.205 | 0.95 | 1.22 |
| **Initial breastfeeding** |  |  |  |  |  |
| Yes Vs No^R^ | 1.00 | 0.066 | 0.900 | 0.88 | 1.14 |
| **Hosmer and Lemeshow Test** | | Chi-square value=5.843; p-value=0.665 | | | |
| **Nagelkerke R^2^ –value** | | 0.56 | | | |

| **Cluster level variation** | 0.0280 |
| --- | --- |

**Supplementary Table 2:** Two-level binary logistic regression analysis of maternal child marriage influencing wasting of under-five children

| **Variable** | **OR** | **SE** | **P-value** | **95% CI for OR** | |
| --- | --- | --- | --- | --- | --- |
|  |  |  |  | **Lower** | **Upper** |
| **Age at first marriage (year)** | | | |  |  |
| Non-child marriage Vs Child marriage^R^ | 1.519 | 0.214 | 0.003 | 1.15 | 2.00 |
| **Division** | | | |  |  |
| Barisal Vs Sylhet^R^ | 0.980 | 0.208 | 0.927 | 0.64 | 1.48 |
| Chittagong Vs Sylhet^R^ | 0.888 | 0.165 | 0.523 | 0.61 | 1.27 |
| Dhaka Vs Sylhet^R^ | 0.922 | 0.182 | 0.683 | 0.62 | 1.35 |
| Khulna Vs Sylhet^R^ | 0.742 | 0.172 | 0.202 | 0.47 | 1.17 |
| Mymensingh Vs Sylhet^R^ | 0.949 | 0.190 | 0.795 | 0.64 | 1.40 |
| Rajshahi Vs Sylhet^R^ | 0.675 | 0.160 | 0.099 | 0.42 | 1.07 |
| Rangpur Vs Sylhet^R^ | 0.819 | 0.181 | 0.370 | 0.53 | 1.26 |
| **Type of place of residence** | | | |  |  |
| Urban Vs Rural^R^ | 1.182 | 0.146 | 0.176 | 0.92 | 1.50 |
| **Mothers’ education level** | | | |  |  |
| No education Vs Higher^R^ | 2.071 | 0.570 | 0.008 | 1.20 | 3.55 |
| Primary Vs Higher^R^ | 1.333 | 0.300 | 0.202 | 0.85 | 2.07 |
| Secondary Vs Higher^R^ | 1.415 | 0.275 | 0.075 | 0.96 | 2.07 |
| **Husbands’ education level** | | | |  |  |
| No education Vs Higher^R^ | 1.260 | 0.302 | 0.334 | 0.78 | 2.01 |
| Primary Vs Higher^R^ | 0.897 | 0.192 | 0.613 | 0.58 | 1.36 |
| Secondary Vs Higher^R^ | 1.09 | 0.217 | 0.632 | 0.74 | 1.62 |
| **Religion** | | | |  |  |
| Muslim Vs Others^R^ | 1.236 | 0.261 | 0.315 | 0.81 | 1.87 |
| **Wealth Index** | | | |  |  |
| Poor Vs Rich^R^ | 1.115 | 0.166 | 0.461 | 0.83 | 1.49 |
| Middle Vs Rich^R^ | 0.963 | 0.158 | 0.819 | 0.69 | 1.33 |
| **Total ever born children** | | | |  |  |
| One Vs Three or more^R^ | 1.448 | 0.209 | 0.010 | 1.09 | 1.92 |
| Two Vs Three or more^R^ | 1.222 | 0.163 | 0.132 | 0.94 | 1.58 |
| **Age at first birth (Year)** | | | |  |  |
| Early child bearing (age<20) Vs Normal (age≥20)^R^ | 1.625 | 0.24 | 0.001 | 1.21 | 2.18 |
| **Husbands’ occupation** | | | |  |  |
| Hard working Vs Unemployed^R^ | 1.270 | 0.464 | 0.513 | 0.62 | 2.60 |
| Service holder Vs Unemployed^R^ | 0.748 | 0.356 | 0.543 | 0.29 | 1.90 |
| Businessman Vs Unemployed^R^ | 0.856 | 0.328 | 0.687 | 0.40 | 1.81 |
| **Respondent currently working** | | | |  |  |
| No Vs Yes^R^ | 0.993 | 0.115 | 0.958 | 0.79 | 1.24 |
| **Sex of child** |  |  |  |  |  |
| Boy Vs Girl^R^ | 1.199 | 0.126 | 0.084 | 0.97 | 1.47 |
| **Initial breastfeeding** |  |  |  |  |  |
| Yes Vs No^R^ | 0.982 | 0.107 | 0.874 | 0.79 | 1.21 |
| **Hosmer and Lemeshow Test** | | Chi-square value=3.999; p-value=0.857 | | | |
| **Nagelkerke R^2^ –value** | | 0.49 | | | |

| **Cluster level variation** | 0.025404 |
| --- | --- |

**Supplementary Table 3:** Two-level binary logistic regression analysis of maternal child marriage influencing underweight of under-five children

| **Variable** | **OR** | **SE** | **P-value** | **95% CI for OR** | |
| --- | --- | --- | --- | --- | --- |
|  |  |  |  | **Lower** | **Upper** |
| **Age at first marriage (year)** | | | |  |  |
| Non-child marriage Vs Child marriage^R^ | 1.150 | 0.118 | 0.047 | 1.09 | 1.82 |
| **Division** | | | |  |  |
| Barisal Vs Sylhet^R^ | 0.636 | 0.099 | 0.004 | 0.46 | 0.86 |
| Chittagong Vs Sylhet^R^ | 0.668 | 0.090 | 0.003 | 0.51 | 0.87 |
| Dhaka Vs Sylhet^R^ | 0.512 | 0.077 | 0.000 | 0.38 | 0.68 |
| Khulna Vs Sylhet^R^ | 0.595 | 0.097 | 0.002 | 0.43 | 0.82 |
| Mymensingh Vs Sylhet^R^ | 0.821 | 0.117 | 0.169 | 0.62 | 1.08 |
| Rajshahi Vs Sylhet^R^ | 0.593 | 0.097 | 0.001 | 0.43 | 0.81 |
| Rangpur Vs Sylhet^R^ | 0.649 | 0.101 | 0.006 | 0.47 | 0.88 |
| **Type of place of residence** | | | |  |  |
| Urban Vs Rural^R^ | 1.131 | 0.103 | 0.178 | 0.94 | 1.35 |
| **Mothers’ education level** | | | |  |  |
| No education Vs Higher^R^ | 2.384 | 0.478 | 0.000 | 1.60 | 3.53 |
| Primary Vs Higher^R^ | 1.806 | 0.295 | 0.000 | 1.31 | 2.48 |
| Secondary Vs Higher^R^ | 1.592 | 0.232 | 0.001 | 1.19 | 2.11 |
| **Husbands’ education level** | | | |  |  |
| No education Vs Higher^R^ | 1.550 | 0.273 | 0.013 | 1.09 | 2.19 |
| Primary Vs Higher^R^ | 1.506 | 0.239 | 0.010 | 1.10 | 2.05 |
| Secondary Vs Higher^R^ | 1.266 | 0.192 | 0.122 | 0.93 | 1.70 |
| **Religion** | | | |  |  |
| Muslim Vs Others^R^ | 0.982 | 0.138 | 0.900 | 0.74 | 1.29 |
| **Wealth Index** | | | |  |  |
| Poor Vs Rich^R^ | 1.360 | 0.143 | 0.004 | 1.10 | 1.67 |
| Middle Vs Rich^R^ | 1.387 | 0.157 | 0.004 | 1.11 | 1.73 |
| **Total ever born children** | | | |  |  |
| One Vs Three or more^R^ | 0.967 | 0.097 | 0.744 | 0.79 | 1.17 |
| Two Vs Three or more^R^ | 0.890 | 0.079 | 0.194 | 0.74 | 1.06 |
| **Age at first birth (Year)** | | | |  |  |
| Early child bearing (age<20) Vs Normal (age≥20)^R^ | 1.196 | 0.123 | 0.081 | 0.97 | 1.46 |
| **Husbands’ occupation** | | | |  |  |
| Hard working Vs Unemployed^R^ | 0.764 | 0.180 | 0.256 | 0.48 | 1.21 |
| Service holder Vs Unemployed^R^ | 0.689 | 0.220 | 0.244 | 0.36 | 1.28 |
| Businessman Vs Unemployed^R^ | 0.597 | 0.148 | 0.038 | 0.36 | 0.97 |
| **Respondent currently working** | | | |  |  |
| No Vs Yes^R^ | 0.968 | 0.077 | 0.690 | 0.82 | 1.13 |
| **Sex of child** |  |  |  |  |  |
| Boy Vs Girl^R^ | 1.139 | 0.082 | 0.072 | 0.98 | 1.31 |
| **Initial breastfeeding** |  |  |  |  |  |
| Yes Vs No^R^ | 0.983 | 0.074 | 0.823 | 0.84 | 1.14 |
| **Hosmer and Lemeshow Test** | | Chi-square value=6.793; p-value= 0.559 | | | |
| **Nagelkerke R^2^ –value** | | 0.60 | | | |

| **Cluster level variation** | 0.0752 |
| --- | --- |
